# Estimating Prevalence, Demographics, and Costs of ME/CFS Using Large Scale Medical Claims Data and Machine Learning

**DOI:** 10.3389/fped.2018.00412

**Published:** 2019-01-08

**Authors:** Ashley R. Valdez, Elizabeth E. Hancock, Seyi Adebayo, David J. Kiernicki, Daniel Proskauer, John R. Attewell, Lucinda Bateman, Alfred DeMaria, Charles W. Lapp, Peter C. Rowe, Charmian Proskauer

**Affiliations:** ^1^Optum Enterprise Analytics, UnitedHealth Group, Minneapolis, MN, United States; ^2^Optum Technology, UnitedHealth Group, Minneapolis, MN, United States; ^3^Innovation, Research, and Development, UnitedHealth Group, Minneapolis, MN, United States; ^4^Bateman Horne Center, Salt Lake City, UT, United States; ^5^Bureau of Infectious Disease and Laboratory Sciences, Massachusetts Department of Public Health, Boston, MA, United States; ^6^Hunter-Hopkins Center, Charlotte, NC, United States; ^7^Children's Center Chronic Fatigue Clinic, Johns Hopkins University School of Medicine, Baltimore, MD, United States; ^8^Massachusetts ME/CFS & FM Association, Quincy, MA, United States

**Keywords:** ME/CFS, myalgic encephalomyelitis, chronic fatigue syndrome, prevalence, costs, machine learning, data mining

## Abstract

Techniques of data mining and machine learning were applied to a large database of medical and facility claims from commercially insured patients to determine the prevalence, gender demographics, and costs for individuals with provider-assigned diagnosis codes for myalgic encephalomyelitis (ME) or chronic fatigue syndrome (CFS). The frequency of diagnosis was 519–1,038/100,000 with the relative risk of females being diagnosed with ME or CFS compared to males 1.238 and 1.178, respectively. While the percentage of women diagnosed with ME/CFS is higher than the percentage of men, ME/CFS is not a “women's disease.” Thirty-five to forty percent of diagnosed patients are men. Extrapolating from this frequency of diagnosis and based on the estimated 2017 population of the United States, a rough estimate for the number of patients who may be diagnosed with ME or CFS in the U.S. is 1.7 million to 3.38 million. Patients diagnosed with CFS appear to represent a more heterogeneous group than those diagnosed with ME. A machine learning model based on characteristics of individuals diagnosed with ME was developed and applied, resulting in a predicted prevalence of 857/100,000 (*p* > 0.01), or roughly 2.8 million in the U.S. Average annual costs for individuals with a diagnosis of ME or CFS were compared with those for lupus (all categories) and multiple sclerosis (MS), and found to be 50% higher for ME and CFS than for lupus or MS, and three to four times higher than for the general insured population. A separate aspect of the study attempted to determine if a diagnosis of ME or CFS could be predicted based on symptom codes in the insurance claims records. Due to the absence of specific codes for some core symptoms, we were unable to validate that the information in insurance claims records is sufficient to identify diagnosed patients or suggest that a diagnosis of ME or CFS should be considered based solely on looking for presence of those symptoms. These results show that a prevalence rate of 857/100,000 for ME/CFS is not unreasonable; therefore, it is not a rare disease, but in fact a relatively common one.

## Introduction

Myalgic Encephalomyelitis (ME) and Chronic Fatigue Syndrome (CFS) are serious, debilitating conditions that impose a burden of illness on millions of people in the United States and around the world (1).

Multiple case definitions have been used to define ME and CFS. Those for ME require the presence of post-exertional malaise and tend to identify a more severely ill subset of the broader ME and CFS population (2). Although there are separate diagnostic codes for ME and CFS, the descriptions in the International Classification of Diseases (ICD)^1^ listings are the same. The two terms ME and CFS have been conflated, and as of 2016, U.S. federal health agencies have used the combined term ME/CFS to refer to this disease.

ME/CFS is an acquired, chronic, multi-systemic disease characterized by significant relapse after physical, cognitive, or emotional exertion of any sort. The disease includes immune, neurological and cognitive impairment, sleep abnormalities, and autonomic dysfunction, resulting in significant functional impairment accompanied by a pathological level of fatigue. The cause of the disease remains unknown, although in many cases symptoms may have been triggered by an infection or other prodromal event [U.S. Department of Health and Human Services, (3)].

The underlying etiology is not known. There is no readily available laboratory test to diagnose ME/CFS, no FDA-approved drug for ME/CFS, and no cure. Many ME/CFS patients experience significant disability. At least one-quarter of ME/CFS patients are house- or bedbound at some point in their lives (4, 5). The direct and indirect economic costs of ME/CFS to society have been estimated at $17 to $24 billion annually (6), including $9.1 billion attributed to lost household and labor force productivity (7).

Assigning a diagnosis of ME/CFS in the clinical setting often takes years. Many physicians are uninformed or misinformed about the disease (1). It has been estimated that 84–91% of patients affected by ME/CFS are not diagnosed with the disease (8).

Thus, improving diagnosis and optimizing management can have significant economic and public health consequences (2). Without good data on the prevalence of ME/CFS, it is difficult to allocate resources for research of all kinds (etiology, pathophysiology, treatment, etc.), as well as for medical education, that would be commensurate with the burden of the disease.

This study uses data from a large sample of the general population insured by a major commercial health insurance carrier to look at characteristics of clinician-diagnosed ME and CFS patients. We applied techniques of data mining and machine learning to a large medical claims database to investigate the prevalence, characteristics, and costs for individuals with ME/CFS.

## Methods

**Figure 1:**
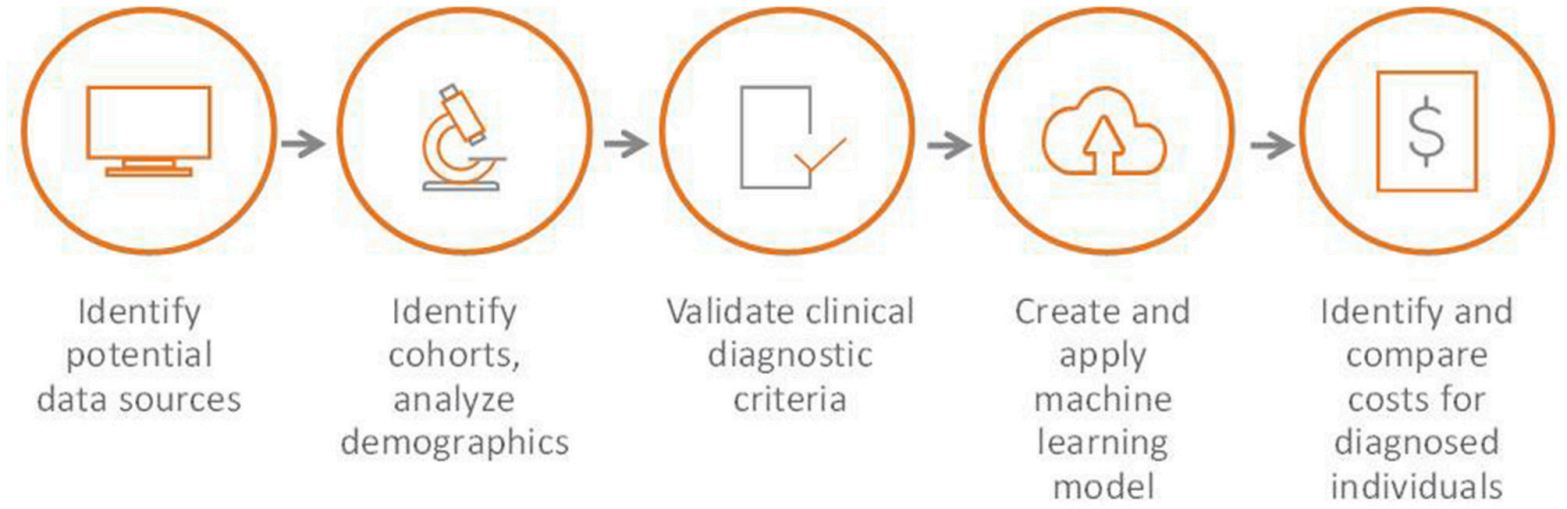
Graphic showing sequence of steps in this study.

This study examined de-identified physician and hospital data from a large claims processing database from Optum, a large healthcare information and services company, which allowed us to describe features of physician-diagnosed ME/CFS patients and to compare this group to the general insured population. The overall sequence of the study is shown in Figure 1.

### Data Sources

The Optum database contains membership, provider, claims, and ancillary data on over 101 million former and current members. The database contains no identifying information on individuals (names, addresses, etc.), but each individual's claims data are linked. The database contains a primary diagnosis and up to four additional diagnostic codes for each claim.

The primary dataset used in this study includes medical and facility insurance claims for nearly 50 million (49,963,500) individuals age 0 through 89 who had at least one medical or one facility claim. Only medical (e.g., from doctor's offices and including any tests or procedures that were conducted or ordered there) and facility (e.g., hospital) claims were part of the data sets used in this study. Prescription drug claims were not included.

The database captured all medical and facility claims, and did not require that the individual have continuous insurance coverage over a specific period of time. The data used in this study are primarily from individuals enrolled during the years 2011–2016, 2016 being the last year for which complete enrollment data were available. All claims for these individuals were considered including claims from the year 2017 if available.

Approximately 80% of individuals in this dataset were insured by commercial health insurance; close to 20% had coverage from Medicare, the U.S. government program for individuals age 65 and over and for certain individuals with disabilities. The dataset did not include any claims associated with Medicaid, the U.S. government program for low-income individuals. For some of the topics in this study, sub-sets of the dataset were used.

We used diagnosis codes which were assigned and entered into the patient's record. From the code alone we could not determine what case definition or clinical diagnostic criteria were used to make the diagnosis.

During the years 2011–2016 two different sets of ICD codes^2^ were in use, ICD-9-CM and ICD-10-CM. Both versions included codes that were used for CFS (Chronic Fatigue Syndrome) and ME (Myalgic Encephalomyelitis) (Table 1). ME and CFS were analyzed separately in some analyses. The lack of specificity and interrelationships of these codes (see Appendix 1 in Supplementary Material, Interrelationships of ICD codes used for Chronic Fatigue Syndrome (CFS) and Myalgic Encephalomyelitis (ME) in the U.S. as of October, 2018) introduce a degree of uncertainty; however, these are the diagnostic codes used for ME and CFS, and provide the best available baseline for this type of study at this point in time.

**Table 1:** ICD codes used for diagnosis of ME and CFS.

**Diagnosis**	**ICD-9-CM (retired Oct 1, 2015)**	**ICD-10-CM**
ME (Myalgic Encephalomyelitis)	323.9	G93.3
CFS (Chronic Fatigue Syndrome)	780.71	R53.82

### Frequency of ME/CFS Diagnosis and Demographics of Diagnosed Population

Main dataset: The general population (all individuals in the database) and the diagnosed population (all those with codes for ME or CFS) were examined for distribution by current (as of 2017) age and gender. Queries were run against the entire data set and two subsets. Separate queries were run to eliminate duplications if an individual had codes for both ME and CFS.

The initial analysis was conducted using the entire dataset of 49,963,500 individuals who had at least one medical or one facility claim. Claims associated with these individuals were examined for presence of one of the four diagnosis codes as the primary diagnosis. Length of insurance enrollment was not considered for this group.

Subset 1: The first subset analysis examined individuals who were continuously enrolled in the same insurance plan for the entire 2011–2016 period, providing longer length of enrollment, and more complete medical history.

Subset 2: The second subset consisted of individuals who were continuously enrolled in the same insurance plan for between 2 and 4 years at any time between 2011 and 2016. This group more accurately represents the central tendencies of the data and eliminates outliers. For this query we used codes from all 5 diagnosis code fields in the analysis, not just the primary diagnosis. All claims for these individuals were included. In addition to the base query, we also separately analyzed demographic data for individuals in this subset who were diagnosed with ME only, without including those diagnosed with CFS alone.

For all datasets, the reference population is all the individuals in that dataset.

### Validation of Clinical Diagnostic Criteria

An important component of this study was an examination of symptom codes in medical records to determine if the diagnosis of ME/CFS could be confirmed by the presence of a unique cluster of symptoms, such as those in the diagnostic criteria proposed in the 2015 report from the National Academy of Medicine (1), which requires 4 or 5 core symptoms that were determined to be strongly supported by evidence as accurately identifying ME/CFS (Appendix 2 in Supplementary Material).

In order to maximize the probability of being able to identify symptoms, we limited analysis to the population continuously enrolled for the entire 2011–2016 period (subset 1), since longer enrollment provides a more complete history.

### Estimating Prevalence Using Machine Learning

We applied the techniques of machine learning to predict the prevalence of ME using another method of analyzing our claims data. (For more information about machine learning, see Appendix 3 in Supplementary Material). From the cohort of individuals who were continuously enrolled for 2 to 4 years, individuals under the age of 15 years were removed in order to minimize features that would be created from predominantly pediatric care. To create the machine learning modeling cohort, we included all members of the diagnosed population as well as a random sample of 25% from the remaining general population.

The modeling cohort was randomly split into a training, validation, and testing set per data science protocol. The training set was rebalanced for modeling with a 50–50 random split, so that the diagnosed and general population were evenly split and the model could train on positive and negative classes evenly. The training set was used to train the model. The validation set was used to tune the model. The testing set was used to do a final test on the finished model.

The model was built using XGBoost, an open source implementation of the boosted tree method of supervised learning. The final model contained 507 features which included medical claim codes, age, and gender information. The validation set was used for prevalence estimates. Prevalence was estimated from individuals that the model predicted to have ME at a 99% probability and dividing by the total number in the dataset.

### Costs

To analyze the financial impact of the disease, we used data from the main data set for the years 2012 through 2016. This time period was chosen because it includes the largest number of individuals and the most years of claims data. We focused on individuals with the ME diagnosis code because we speculated that assignment of the less well-known ME diagnosis code might better represent the characteristics and diagnostic criteria of ME/CFS, and therefore this would be a more specific group. We only considered individuals from the overall cohort who were 13 years of age or older, since the incidence of ME in young children is much smaller.

Costs used were the standard allowed payment (contracted rate) for all provider services which may have ultimately been paid by either the insurer or related patient responsibility associated with the claim such as patient co-payment or deductible, if any.

We looked at the yearly costs related to claims for individuals diagnosed with ME vs. all other individuals in the reference population. The average annual cost per individual was calculated on medical claims >$0 for each year from 2012 to 2016. Costs were not adjusted for inflation. Costs included both those paid by the insurer at the standard allowed payment for all provider services and the related patient responsibility associated with the claim such as patient co-payment or deductible, if any. Only medical and facility claims were considered, and we did not analyze the content of claims that contributed to the costs.

To put the cost in context, we also looked in the same way at annual costs related to claims for two similar diseases which are often compared to ME/CFS, multiple sclerosis and lupus erythematosus. ICD codes used in these queries are shown in Table 2.

**Table 2:** ICD codes used for the diagnosis of multiple sclerosis and lupus.

**Diagnosis**	**ICD-9-CM (retired Oct 1, 2015)**	**ICD-10-CM**
MS (Multiple Sclerosis)	340	G35
Lupus (includes all subcategories)	710	M32, L93

## Results

### Prevalence of ME Diagnosis vs. Average Length of Enrollment

In creating Subset 2, we compared the average length of continuous enrollment for individuals diagnosed with ME-only vs. the general insured population. The average length of enrollment of all individuals in the database is just over 2 years (980 days). Individuals with a diagnosis code for ME but not for CFS, on average, have been enrolled for just over 3 years (1,204 days). We therefore chose the length of continuous enrollment from 2 to 4 years to most accurately represent the central tendencies of the data and eliminate outliers for both the general and the ME-diagnosed population. Subset two includes individuals with a diagnosis of ME in any of the diagnosis fields in the claims (primary diagnosis plus up to four additional secondary diagnoses).

Table 3 shows the number of individuals continuously enrolled for periods of from 1 to 7 years who had a diagnosis code of ME (but not CFS). Note that as the length of continuous enrollment increases, the proportion with an ME diagnosis also increases, as would be expected as the opportunity for diagnosis is extended.

**Table 3:** Prevalence of ME diagnosis for varying lengths of continuous enrollment (any years).

**Continuous years enrolled**	**Diagnosed**	**General Pop**.	**Prevalence per 100,000**
0–1	2,668	3,648,421	73
1–2	11,070	13,422,797	82
2–3	7,883	7,339,562	107
3–4	6,337	4,438,630	143
4–5	6,375	3,660,868	174
5–6	2,971	1,670,694	178
6–7	4,925	2,629,342	187
Total	42,229	36,810,314	115

### Frequency of ME/CFS Diagnosis

Tables 4, 5 show diagnostic codes for ME and CFS and prevalence of these diagnoses for the three population sets. Data columns for ME and CFS include all individuals who had that as the primary diagnosis code in any of their claims. Prevalence for each of the three groups was calculated by dividing the number of diagnosed individuals by the total in the reference population. Some individuals might have had both codes within their set of claims; separate queries were run to eliminate this duplication. Table 4 shows the prevalence for ME and CFS separately. Table 5 shows the prevalence for ME+CFS with and without duplication. Without duplication, prevalence of ME/CFS was 519/100,000 in the main dataset (non-continuous enrollment), 669/100,000 in Subset 1 (continuous enrollment for the entire period), and 1,038/100,000 in Subset 2 (continuous enrollment for any 2–4 year period). For Subset 2 only, up to four secondary diagnosis fields were included from the claims in addition to the primary diagnosis.

**Table 4:** Summary of prevalence of ME and CFS in three studied cohorts.

**Population**	**ME**				**CFS**			
	**G93.3**	**323.9**	**Total w/Dups**	**per 100K**	**R53.82**	**780.71**	**Total w/dups**	**per 100K**
Main dataset	16,305	9,263	25,568	51	140,947	99,929	240,876	482
Subset 1	1,044	1,030	2,074	81	6,635	10,234	16,869	661
Subset 2	10,196	3,945	14,141	121	87,282	57,614	144,896	1,236

**Table 5:** Summary of prevalence of ME + CFS in the three studied cohorts, and with duplicates eliminated.

**Population**	**ME+CFS**		**Union ME+CFS**		**Reference**
	**Total**	**per 100K**	**Total no dups**	**per 100K**	**Total**
Main dataset	266,444	533	259,275	519	49,963,500
Subset 1	18,943	742	17,074	669	2,553,722
Subset 2	159,037	1,357	121,632	1,038	11,720,401

Extrapolating from this frequency of diagnosis and based on the estimated 2017 population of the United States of 325,719,178 (9), a rough estimate for the number of patients who are diagnosed with ME or CFS in the U.S. is 1.7 million to 3.4 million.

### Demographics of Diagnosed Population

**Figure 2:**
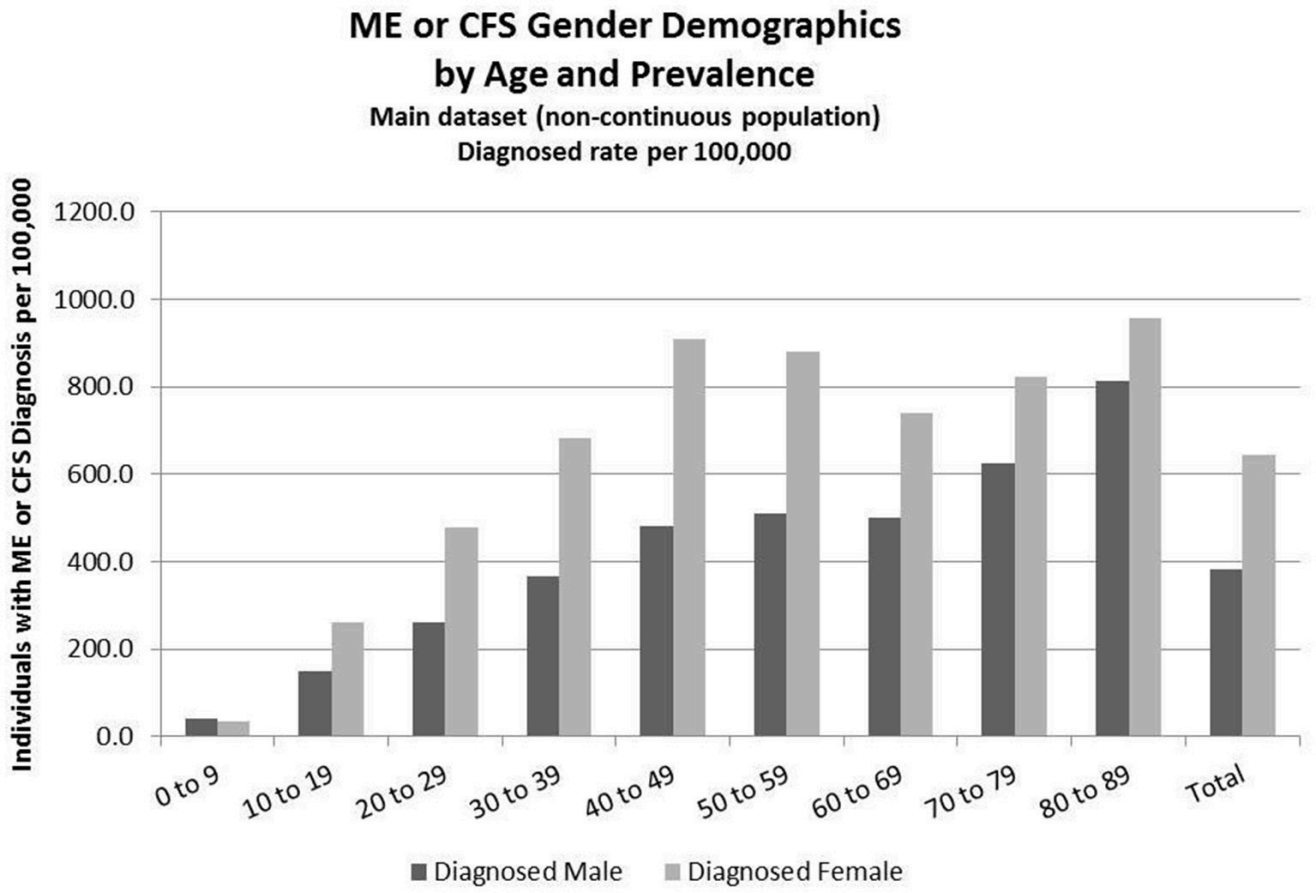
ME or CFS gender demographics by age and prevalence (main dataset, non-continuous enrollment).

**Figure 3:**
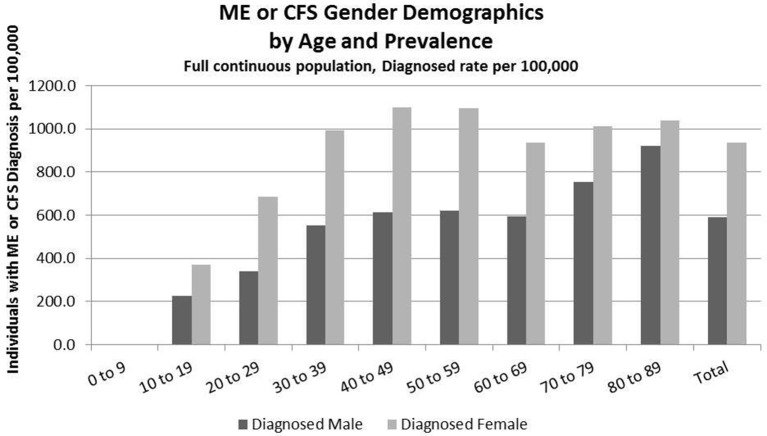
ME or CFS gender demographics by age and prevalence (Subset 1, continuous enrollment from 2011 to 2016).

**Figure 4:**
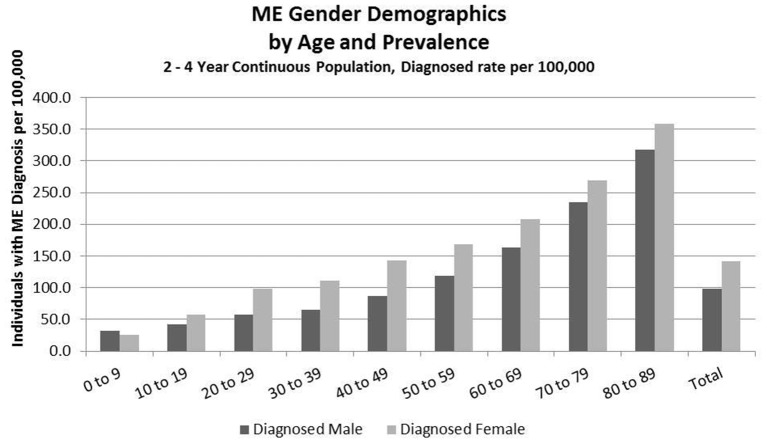
ME gender demographics by age and prevalence (Subset 2, continuous enrollment from 2 to 4 years).

Detailed analysis of gender distribution by age for ME or CFS diagnosed individuals (no duplicates) within the three studied population sets are shown in Figures 2–4 and Tables 6, 7. Totals for the gender distribution are slightly smaller because gender information was not available for every individual. Results are normalized for each decile.

**Table 6:** ME or CFS gender demographics by age and prevalence vs. reference population in the main dataset (Main dataset: non-continuous enrollment).

	**Diagnosed-ME/CFS (count/100,000)**			**F: M ratio**	**Diagnosed-ME/CFS (% normalized)**		**Diagnosed-ME/CFS (count)**			**Data set population**		
**Age range**	**M**	**F**	**Total**	**F: M**	**M**	**F**	**M**	**F**	**Total**	**M**	**F**	**Total**
0 to 9	40.0	34.3	37.2	0.86: 1	53.83%	46.17%	978	796	1,774	2,446,920	2,321,941	4,768,861
10 to 19	148.8	261.8	204.8	1.76: 1	36.24%	63.76%	4,023	6,934	10,957	2,702,876	2,648,306	5,351,182
20 to 29	259.5	478.1	379.3	1.84: 1	35.18%	64.82%	7,725	17,245	24,970	2,976,670	3,606,744	6,583,414
30 to 39	365.8	681.9	539.0	1.86: 1	34.91%	65.09%	11,679	26,390	38,069	3,192,640	3,869,894	7,062,534
40 to 49	482.1	909.1	708.4	1.89: 1	34.65%	65.35%	15,264	32,472	47,736	3,166,430	3,571,767	6,738,197
50 to 59	510.4	879.5	705.2	1.72: 1	36.72%	63.28%	16,865	32,459	49,324	3,304,031	3,690,758	6,994,789
60 to 69	499.6	739.6	628.1	1.48: 1	40.31%	59.69%	14,433	24,610	39,043	2,889,185	3,327,290	6,216,475
70 to 79	623.7	822.8	731.3	1.32: 1	43.12%	56.88%	9,976	15,479	25,455	1,599,520	1,881,168	3,480,688
80 to 89	814.2	958.0	900.3	1.18: 1	45.94%	54.06%	7,766	13,608	21,374	953,817	1,420,412	2,374,229
Total	381.8	645.4	521.9	1.69: 1	37.17%	62.83%	88,709	169,993	258,702	23,232,089	26,338,280	49,570,369

**Table 7:** ME or CFS gender demographics by age and prevalence vs. reference population (Subset 1: continuous enrollment 2011–2016).

	**Diagnosed-ME/CFS (count/100,000)**			**F: M ratio**	**Diagnosed-ME/CFS (% normalized)**		**Diagnosed-ME/CFS (count)**			**Data set population**		
**Age range**	**M**	**F**	**Total**	**F: M**	**M**	**F**	**M**	**F**	**Total**	**M**	**F**	**Total**
0–9	0.0	0.0	0.0		0.00%	0.00%	0	0	0	4	5	9
10 to 19	179.7	288.7	233.1	1.61: 1	38.36%	61.64%	242	374	616	134,694	129,563	264,257
20–29	297.3	584.0	443.8	1.96: 1	33.74%	66.26%	405	831	1,236	136,205	142,303	278,508
30–39	481.6	881.7	695.9	1.83: 1	35.33%	64.67%	270	570	840	56,062	64,646	120,708
40–49	515.3	956.9	749.2	1.86: 1	35.00%	65.00%	780	1,631	2,411	151,354	170,449	321,803
50–59	530.7	947.4	749.4	1.79: 1	35.91%	64.09%	1,155	2,277	3,432	217,625	240,347	457,972
60–69	527.7	819.9	682.5	1.55: 1	39.16%	60.84%	1,073	1,877	2,950	203,345	228,921	432,266
70–79	640.2	848.8	752.9	1.33: 1	43.00%	57.00%	695	1,082	1,777	108,557	127,478	236,035
80–89	791.5	909.7	862.8	1.15: 1	46.52%	53.48%	1,385	2,420	3,805	174,985	266,008	440,993
Total	507.7	807.6	668.6	1.59: 1	38.60%	61.40%	6,005	11,062	17,067	1,182,831	1,369,720	2,552,551

### Demographics of Diagnosed Population for the Main Dataset (Non-continuous Enrollment)

Figure 2 and Table 6 show the gender distribution by age for individuals diagnosed with ME or CFS for the population of individuals who were enrolled at any time during the period 2011–2016, and the gender distribution by age and prevalence for these same individuals.

Of the 49,570,369 individuals enrolled during the period 2011–2016 for whom we have gender information, 258,702 (or 519/100,000) had a code for diagnosis of either CFS or ME. The relative risk for females being diagnosed with ME or CFS compared to males was 1.238 (95% CI: 1.235–1.242).

In the youngest age group, 0–9, boys outnumber girls; relative risk for females being diagnosed with ME or CFS compared to males in this age group was 0.922 (95% CI: 0.874–0.970).

### Demographics of Diagnosed Population, Subset 1, Continuous Enrollment From 2011 to 2016

Figure 3 and Table 7 show the gender distribution by age for individuals diagnosed with ME or CFS for the population of individuals who were continuously enrolled in their insurance for the entire period 2011–2016 (Subset 1) and the gender distribution by age and prevalence for these same individuals. In this group there were no diagnosed individuals younger than 10.

Of the 2,552,551 individuals continuously enrolled for the entire period 2011–2016 for whom we have gender information, 17,067 (669/100,000) have a code for diagnosis of either CFS or ME; relative risk for females being diagnosed with ME or CFS compared to males was 1.210 (95% CI: 1.196–1.223).

### Demographics of Diagnosed Population, Subset 2, Continuous Enrollment 2 to 4 Years

Figure 4 and Table 8 show the gender distribution by age for individuals enrolled for a period of from 2 to 4 years and having a diagnosis code of ME in any diagnosis field in the claim, and the gender distribution by age and prevalence for these same individuals. The overall prevalence of a diagnosis of ME only (no CFS diagnosis) in the cohort continuously enrolled for 2 to 4 years is 121/100,000. The relative risk for females being diagnosed with ME compared to males was 1.178 (95% CI: 1.162–1.194). In the youngest age group, 0–9, boys outnumber girls once again.

**Table 8:** ME gender demographics by age and prevalence vs. reference population (Subset 2, continuous enrollment 2 to 4 years).

	**Diagnosed-ME (count/100,000)**			**F: M ratio**	**Diagnosed-ME (% normalized)**		**Diagnosed-ME (count)**			**Data set population**		
**Age range**	**M**	**F**	**Total**	**F: M**	**M**	**F**	**M**	**F**	**Total**	**M**	**F**	**Total**
0–9	32.7	25.9	29.4	0.79: 1	55.81%	44.19%	207	157	364	633,270	606,551	1,239,821
10–19	42.0	58.1	50.0	1.38: 1	41.97%	58.03%	307	412	719	730,324	708,729	1,439,053
20–29	57.4	97.8	77.5	1.70: 1	37.00%	63.00%	462	778	1,240	804,286	795,311	1,599,597
30–39	65.8	111.1	88.6	1.69: 1	37.20%	62.80%	545	932	1,477	827,930	838,616	1,666,546
40–49	86.9	142.5	114.7	1.64: 1	37.89%	62.11%	668	1,099	1,767	768,545	771,386	1,539,931
50–59	118.5	168.9	144.1	1.43: 1	41.22%	58.78%	903	1,335	2,238	762,241	790,364	1,552,605
60–69	164.0	207.6	187.0	1.27: 1	44.13%	55.87%	1,090	1,540	2,630	664,725	741,738	1,406,463
70–79	234.3	268.7	253.1	1.15: 1	46.58%	53.42%	824	1,135	1,959	351,709	422,440	774,149
80–89	317.9	358.1	342.5	1.13: 1	47.02%	52.98%	621	1,099	1,720	195,363	306,871	502,234
Total	98.1	141.9	120.4	1.45: 1	40.87%	59.13%	5,627	8,487	14,114	5,738,393	5,982,006	11,720,399

### Validation of Clinical Diagnostic Criteria

In developing the list of appropriate symptom codes (Appendix 4 in Supplementary Material) it became apparent that existing codes do not fully identify symptoms that specifically describe ME/CFS. Most importantly, there is no symptom code specifically for post-exertional malaise, a core symptom, and codes for various types of fatigue do not match well with the description of another of the core symptoms, i.e., a substantial level of impairment in the ability to engage in pre-illness activities accompanied by fatigue.

**Figure 5:**
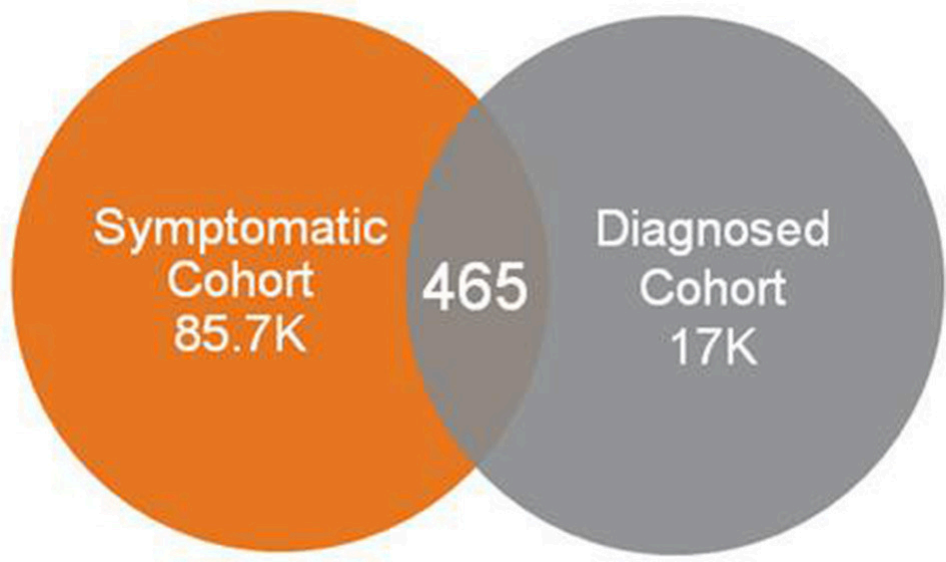
Overlap of individuals with some symptoms of ME/CFS vs. those diagnosed.

Use of symptom codes relating to fatigue, sleep abnormalities, cognitive impairment and orthostatic intolerance, and without requiring codes possibly representing post-exertional malaise from consideration, resulted in a very small number of individuals who were diagnosed with ME or CFS. The vast majority of individuals who had a diagnosis code of ME or CFS did not appear in this symptomatic cohort (Figure 5).

### Machine Learning

We were unable to create a model for ME and CFS together that could be trained and tuned to a sensitivity accuracy of much better than 50%. This indicated that there was insufficient correlation between the input data (features) and outcome (diagnosis of ME or CFS) for the algorithm to make a useful prediction.

After failing to have the model resolve when including common symptom data along (CFS diagnosis), we refocused on diagnosis of ME (diagnosis presumed to include assessment of impaired function and the presence of PEM as core symptoms). The ME model was able to be trained and tuned successfully to achieve sensitivity of 0.738 (95% CI: 0.721–0.754) and specificity of 0.823 (95% CI: 0.823–0.823) with the threshold set at 0.6. The threshold signifies that the model will identify an individual as having ME if they have a risk score greater than or equal to 60%.

Based on the machine learning predictive model, the projected prevalence of ME in our continuously insured population was 857/100,000, calculated from the number of individuals predicted by the model to have an ME diagnosis when the model was evaluated using 99% probability (3,989) and dividing by the total number of individuals in the dataset (465,193). This methodology was used to capture individuals who are undiagnosed, but are most likely to be living with an ME-like illness. The gender distribution, normalized to size of population by gender, was 1.38:1 (58% female and 42% male).

The top predictive features (those with the highest weights) in the model, which included both ICD and CPT codes submitted to insurance, are listed in Table 9.

**Table 9:** Top predictive features for ME machine learning model.

**Score**	**Feature**	**Description**
0.105990	age
0.017413	gender
0.016377	icd_R53	Malaise and fatigue
0.014899	cpt_00175	Qualitative_or_Semiquantitative_Immunoassays
0.014763	icd_N39	Other disorders of urinary system
0.014508	icd_E55	Vitamin D deficiency
0.014083	cpt_00123	Diagnostic_Radiology_(Diagnostic_Imaging)_Procedures_of_the_Head_and_Neck
0.013081	cpt_00124	Diagnostic_Radiology_(Diagnostic_Imaging)_Procedures_of_the_Chest
0.012911	icd_R07	Pain in throat and chest
0.012452	cpt_00128	Diagnostic_Radiology_(Diagnostic_Imaging)_Procedures_of_the_Abdomen
0.011824	icd_R51	Headache
0.011824	cpt_00217	Cardiography_Procedures
0.011178	cpt_00168	Urinalysis_Procedures
0.010957	icd_R06	Abnormalities of breathing
0.010499	icd_R00	Abnormalities of heart beat
0.010465	icd_R94	Abnormal results of function studies
0.010431	cpt_00289	Subsequent_Hospital_Care_Services
0.010074	icd_R50	Fever of other and unknown origin
0.009819	icd_D64	Other anemias
0.009751	icd_E03	Other hypothyroidism
0.009429	cpt_00367	Temporary_National_Codes_(Non-Medicare)
0.009378	cpt_00220	Echocardiography_Procedures
0.008987	icd_K59	Other functional intestinal disorders
0.008885	cpt_00350	Ambulance_and_Other_Transport_Services_and_Support
0.008596	cpt_00174	Hematology_and_Coagulation_Procedures
0.008392	icd_Z51	Encounter for other aftercare and medical care
0.008307	icd_M62	Other disorders of muscle
0.00739	icd_R79	Other abnormal findings of blood chemistry
0.007339	cpt_00160	Diagnostic_Nuclear_Medicine_Procedures
0.007203	icd_R26	Abnormalities of gait and mobility

### Costs

Table 10 shows the average annual medical costs paid by insurance and the patient by year for individuals diagnosed with ME, as well as those diagnosed with lupus or multiple sclerosis, vs. those in the reference population. Costs used were the standard allowed payment (contracted rate) for all provider services which may have ultimately been paid by either the insurer or related patient responsibility associated with the claim such as patient co-payment or deductible, if any.

**Table 10:** Average yearly medical costs for diagnosed vs. reference population.

**Year**	**General population**	**ME**	**Lupus**	**MS**
2016	$ 8,500	$ 30,600	$ 22,600	$ 23,220
2015	$ 7,800	$ 32,400	$ 21,100	$ 22,090
2014	$ 7,500	$ 31,300	$ 20,100	$ 21,050
2013	$ 7,700	$ 34,300	$ 20,100	$ 22,780
2012	$ 7,300	$ 25,700	$ 16,900	$ 19,160
Average	$ 7,760	$ 30,860	$ 20,160	$ 21,660

The average annual medical cost per individual diagnosed with ME in our dataset was $30,860, while the average annual medical cost per individual in the general population in the database was $7,760. For comparison, the average annual cost in our dataset for lupus patients was $20,160 and for multiple sclerosis patients, $21,660.

The costs varied by year, but on average, ME patients had medical costs that were three to four times greater than those in the general population, and ~50% higher than either lupus or multiple sclerosis patients.

## Discussion

Prevalence of ME/CFS has been difficult to estimate due to a number of factors including lack of specific diagnostic tests, multiple case definitions, different methodologies, and confusion about coding. This study offers a new approach to this problem, using a large dataset of insurance claims to examine various characteristics of the group of patients for whom health care providers have given a diagnosis code for CFS or ME. We used a variety of data analysis techniques similar to those used in commercial research, which provide a range of estimates, and compare our results to other methods which have been used to estimate prevalence.

### Coding and Diagnosis Considerations

The diagnostic codes for CFS (in ICD-10-CM) and for ME (in both ICD-10-CM and ICD-9-CM) are not exclusive to these diseases and can include other conditions, which introduces an unknown degree of uncertainty into any prevalence estimates based on these diagnostic codes (see Appendix 1 in Supplementary Material, Interrelationships of ICD codes used for Chronic Fatigue Syndrome (CFS) and Myalgic Encephalomyelitis (ME) in the U.S. as of October, 2018).

A proposal to change the coding for ME, CFS, and related conditions was made at the September 12, 2018 meeting of the National Center for Health Statistics which addresses this ambiguity (10). If this proposal is approved, ME, CFS, Systemic Exercise Intolerance Disease (SEID, the new term recommended by the Institute of Medicine report in 2015), and postviral fatigue syndrome will have separate and distinct codes beginning in October 2019.

For better tracking of this disease, two options could be considered. In the short term, and if the new proposal is not approved, providers who diagnose ME/CFS could use the ICD-10-CM code of G93.3 for ME/CFS and *not* use R53.82 (Chronic Fatigue, unspecified). Ultimately, if the proposed changes are approved, researchers could use the specific codes for the conditions they are tracking.

Diagnosis may vary depending on the case definition or diagnostic criteria used by the provider. Furthermore, there is considerable ongoing investigation on the effect of using different case definitions on the diagnosis of ME and or CFS (11), and this affects whether it is legitimate to use an umbrella term to describe the two conditions (12). While we refer to ME/CFS in this study, our analysis is based on diagnosis of ME and CFS separately, as identified by the specific diagnostic codes for each, although there is no way to know how each medical provider makes a diagnosis and assigned a code.

### Prevalence of ME/CFS Based on Frequency of Diagnosis in an Insurance Claims Database

This is the first study to determine the frequency of ME/CFS diagnosis using insurance claims data for a large number of individuals. Prevalence estimates for chronic fatigue syndrome in the U.S. have been as low as 235/100,000 (373/100,000 in women and 83/100,000 in men) (13) and as high as 2540/100,000 (14). Our prevalence estimates ranged from 519/100,000 to 1,038/100,000 (0.52% to 1.03%), which fall between those expected from large-scale health surveys and smaller scale community-based studies.

Our highest prevalence estimate for ME/CFS, 1,038/100,000 or 1.04%, was found in the group most representative of the insured population—those continuously enrolled for 2 to 4 years—and with the broadest reach: it includes all individuals with ME or CFS as either a primary or a secondary diagnosis, and all claims for these individuals. This estimate can be compared with health surveys conducted in Canada and in some states in the U.S. Recent CDC prevalence estimates of ME/CFS from the Behavioral Risk Factor Surveillance System (BRFSS) (lifetime 1.6%; current 1.2%) (15) were similar to Canadian Community Health Survey 2003 (1.3%) 2010 (1.4%), and 2014 (1.4%) data (16–18). These survey studies do not verify that the specific diagnosis code has been entered in the patient's medical record. They only indicate that the patient is reporting having been given this diagnosis by the health care provider.

Our lowest prevalence estimate for ME/CFS, 519/100,000 or 0.52%, was calculated using all claims from individuals who have ME or CFS as the primary diagnosis and with no restriction on the length of enrollment. This group could therefore have an unknown number of individuals with only one miscellaneous claim, thereby diluting the sample. Nevertheless, the prevalence of ME/CFS in this group is higher than predicted by community-based studies which verified the diagnosis with a medical examination and verifying symptoms using an accepted ME or CFS case definition (e.g., Jason et al., (19), 0.42%; 7, 0.24%; 10, 0.2%).

Our intermediate prevalence estimate has no direct comparisons with previously published results. The prevalence of ME/CFS in the group continuously enrolled for the full 7 years and with ME or CFS as the primary diagnosis, the most restrictive group, is 669/100,000 or 0.67%. Note that this is somewhat higher than the 519/100,000 calculated from the non-continuously enrolled population. The group of individuals continuously enrolled in the same health insurance for a long period of time may include a higher proportion of sicker people than the other groups, but we did not assess this.

Using the diagnosis of ME only, prevalence in the group continuously enrolled for 2 to 4 years is 121/100,000, or 0.12%. This lower prevalence of ME compared with ME/CFS would be expected, as the case definitions for ME are much less well-known by medical providers than CFS. Nevertheless, our sample included more than 14,000 individuals with this diagnosis, which is quite large compared with most studies which examine the characteristics of this group.

Using claims data alone, it is not possible to determine what criteria health care providers are using to make a diagnosis of either ME or CFS. Likewise, a provider might tell a patient they have ME/CFS without the specific diagnostic code being entered into the patient's record.

The prevalence of ME/CFS could be **overestimated** if providers or medical coders are using the CFS diagnosis code to identify a “CFS-like” illness or condition, without reference to any case definition, or simply “chronic fatigue.”

The prevalence of ME/CFS could be **underestimated** if providers or medical coders (a) use a different diagnosis that is less specific (e.g., 780.79, Other Malaise and Fatigue, R53.81 Other Malaise or R53.83 Other Fatigue); (b) do not put ME or CFS diagnosis into the record due to not wanting to expose their patients to a perceived stigma of the disease (1), due to not wanting to provide a discouraging diagnosis when there is no cure, or due to knowing that some appropriate treatments might not be covered under that diagnosis; and (c) if providers are unaware of the diagnosis of ME/CFS, since diagnosis and management of ME/CFS is not taught in most medical schools (20, 21).

It has been reported (8) that 84–91% of patients with ME/CFS are undiagnosed. However, this study is now 14 years old, and so may not reflect the increased awareness of ME/CFS in recent years, which could result in a higher rate of diagnosis. The earlier case definitions used in previous studies require the diagnosis to be one of exclusion, resulting in less likelihood of diagnosis than with more recent clinical diagnostic criteria published by the Institute of Medicine (1).

### Age, Gender, and Prevalence in the Diagnosed Population

This study also shows a lower ratio of females to males diagnosed with ME/CFS than is generally reported. While many studies show a much higher percentage of females (as high as 80% female −7; 9) at least one health survey (17) shows a lower percentage of 65% female. For ME/CFS, our studies show an average of between 60 and 65% female across age groups, except in the youngest group, 0–9 years, where boys outnumber girls. For ME only, the percentage of females is lower, 60%, and again for the youngest group, boys outnumber girls.

While our numbers are based on diagnostic codes in the medical record and reflect actual clinical practice, there is no information about what criteria the providers used to assign these codes, or if they evaluate men differently than women. Nevertheless, the higher than expected number of males with this diagnosis is interesting, and the possible reasons for this need more study.

#### Prevalence in Children and Adolescents

There is little published data on prevalence of ME/CFS in children and adolescents. One community-based study reports a prevalence for adolescents (aged 13 to 17) of 181 per 100,000 or 0.181% (22). Our main dataset shows a prevalence of 37.2/100,000 in children 0–9 years, and 204.5/100,000 in ages 10–19 (Table 6).

### Validation of Clinical Diagnostic Criteria

One goal of the study was to determine whether the presence of specific symptom codes within administrative medical claims data could identify individuals for whom a diagnosis of ME/CFS should be considered. Lack of specific codes for two of five core symptoms required for a diagnosis of ME/CFS using the IOM criteria made it impossible to identify individuals for whom this diagnosis should be considered from administrative claims data, or to validate that individuals diagnosed with ME/CFS had documented evidence of the required core symptoms in their claims data.

### Prevalence Based on Machine Learning

Based on the 2017 population of the U.S. noted earlier, the predicted prevalence rate based on our model of 857/100,000 translates to up to 2.8 million people with ME/CFS in the U.S. This number is somewhat larger than other published estimates of 836,000 to 2.5 million Americans (19) and is significant because it is predicted based on characteristics drawn from those diagnosed with ME only, not including those with a diagnosis of CFS only.

The machine learning technique is a useful way to compensate for the lack of specific symptom codes which might otherwise be used to predict or identify undiagnosed patients. It uses a weighted analysis of a large number of “features” (over 500) derived from a known group (in this case, individuals already diagnosed with ME) to identify individuals with a similar combination of factors. The model can be “tuned” to a desired balance of specificity and sensitivity. Our model performed reasonably well at a threshold of 0.6 (sensitivity 0.82336 and specificity 0.73787). If specific symptom codes for ME were available the model could be improved. To predict the prevalence of ME from our dataset we used a probability threshold of 0.99. Using the 99% probability cut-off is a conservative approach, but provides a reasonable estimate.

The inability to train the machine learning model when CFS diagnoses were included indicates that the population of individuals diagnosed with CFS is too heterogeneous for this method. In contrast, individuals diagnosed with ME were a more homogenous population for which this approach was more effective.

The CFS diagnosis code, in the signs and symptoms section of the ICD, is perhaps being used incorrectly to indicate the symptom of chronic fatigue, which is characteristic of many different underlying conditions, or a “CFS-like” illness which may lack some of the defining features of Chronic Fatigue Syndrome. However, patients coded with ME, which has clinical information identical to Chronic Fatigue Syndrome in ICD-10-CM and is placed in a disease chapter, were significantly, and usefully, more homogeneous. This supports our supposition that clinicians using the ME code are more familiar with the disease than clinicians using the CFS code, and thus may be specifically diagnosing ME/CFS, not using the diagnosis code to cover unspecified chronic fatigue or a “CFS-like” illness.

These results show that the predicted prevalence rate of 857/100,000 based on the machine learning model is not unreasonable for ME/CFS, including the symptom of post-exertional malaise. This estimate suggests that ME/CFS is not a rare disease, but in fact a relatively common one, and offers a new benchmark for future studies.

### Costs

Direct medical costs are important to insurers, who need to deliver good medical care in a cost-effective way, and to patients, who must pay both insurance premiums and out-of-pocket for co-payments, deductibles and treatments that are uninsured.

Direct medical costs for caring for ME/CFS patients are significantly higher than for the general population. Specific components contributing to increased costs (hospitalization, specialist visits, diagnostic tests, presence of other chronic conditions, etc.) were not examined.

Many patients cite a long and costly journey to receiving an accurate diagnosis of ME/CFS (23). Further, once diagnosed, most patients struggle to find primary care providers who are knowledgeable about the condition and well versed in the best practices for managing the symptoms. These twin challenges in diagnosis and treatment are certainly contributors to added cost in the healthcare system.

Direct medical costs are only one component of the total disease costs; others include disability claims, health insurance premiums, and expenses not submitted to insurance such as alternative treatments, nutritional supplements, costs to the economy due to productivity loss, costs to the family for caretaking, and possibly early death. Previous studies have estimated the total annual cost to the economy from ME/CFS to be $17–24 billion (2008 dollars) (6).

Patients with ME/CFS have a high level of disability. Despite high direct medical costs, these patients often have significant unmet health care needs (17) or forgo routine medical care (15). Health surveys have indicated that ME/CFS patients also tend to have more than one chronic condition (15). All these factors could combine and result in poorer quality of life for the patient and even higher medical costs in the future, as well as increasing the burden of illness.

The data from this study illustrate the high costs of the illness, and point to the potential for cost control if patients are diagnosed and provided with the most effective care. Good medical management also holds the promise of improving the experience of patients living with ME/CFS.

### Putting the Results in Context

#### Prevalence

The estimated prevalence of ME/CFS in our study ranges from 519 to 1,038/100,000, and falls between the rates estimated from community health studies and self-reported health surveys. Our study uses larger samples than previous studies, and two different methodologies. Our studies show a range of gender distribution, with the lowest ratio of female to male occurring in the youngest age group, 0–9 years, where boys outnumber girls, and in groups diagnosed with ME only.

Table 11 illustrates the spectrum of prevalence studies which use a variety of techniques. Comparing these studies shows the range of prevalence and gender distribution. Bolded entries are from this study.

**Table 11:** Comparison of prevalence rates.

**Source**	**Population size**	**Prevalence per 100,000**	**% Female**	**Method**
Diagnosed with ME (subset 2, continuous enrollment 2–4 years)	11.7M	121	60.1%	Insurance Claim Data
Nacul et al. (24), (ME/CFS, U.K.)	143,000	200	51.0%	Community Health Study
Reyes et al. (13) (ME/CFS, Wichita, KS)	90,316	240	81.8%	Community Health Study
Jason et al. (19) (ME/CFS, U.S.)	18,675	420	71.9%	Community Health Study
Diagnosed with ME or CFS (main dataset, non-continuous enrollment)	50M	519	65.7%	Insurance Claim Data
Diagnosed with ME or CFS (subset 1, continuous enrollment 2011–2016)	2.5M	669	64.7%	Insurance Claim Data
Projected prevalence of ME using machine learning	2.7M	857	57.9%	Machine Learning Predictive Model
Diagnosed with ME or CFS (subset 2, continuous enrollment 2 to 4 years)	11.7M	1038	65.0%	Insurance Claim Data
National ME/FM Action Network (17) and ME Association of Ontario (16) (Canadian Community Health Surveys)	65,000	1,400	63.4%	Survey
Lin et al. (15) (BRFSS survey, ME/CFS, several states)	54,695	1,600	80.0%	Survey

Generally accepted gender ratios for ME/CFS in the community are as high as 3:1 or 4:1 female to male F (75–80% female). Our data indicate that the actual rate of diagnosis is much less skewed based on gender, though still more commonly diagnosed in women, with a range of 60–65% female.

#### Disease Burden

The World Health Organization has pioneered the use of the Disability Adjusted Life Year (DALY) as a single measure of disease burden in a population (25) and importantly, it includes a measure of the degree of disability from the disease. Using the DALY measure, ME/CFS has been estimated to have a higher total disease burden than multiple sclerosis, autism, or HIV/AIDS (26).

Lupus and multiple sclerosis (MS) are two diseases which are better known than ME/CFS and often compared to it. Although they have different etiologies they have some similar characteristics and symptoms. Both significantly affect quality of life, may take some time to diagnose, affect more women than men, present with some of the same symptoms, and like ME/CFS are often diagnosed late and/or inaccurately initially.

Table 12 compares the estimated prevalence and number of patients in the United States, the burden of illness, and average annual medical cost, and NIH research spending for ME/CFS, lupus, and multiple sclerosis (29, 30). As shown in Table 12, ME/CFS affects more than double the number of persons in the U.S. than lupus and four times as many as MS. The prevalence of lupus is less than half that of ME/CFS, and the prevalence of multiple sclerosis, in a comparable study of commercially-insured patients, is less than one-third of the prevalence of ME/CFS as found in our study.

**Table 12:** Comparison of several factors relating to ME/CFS, lupus and multiple sclerosis.

**Disease**	**# Patients based on est. 2017 U.S. population**	**Prevalence**	**Burden of illness (DALY–disability adjusted life years)**	**Average annual medical cost**	**NIH research spending 2017 (NIH categorical spending, 2017)**
ME/CFS	1,726,000–3,746,000	519–1,038/100,000 0.52–1.04%	714000 (26)	$30,860	$15MM
Lupus	785,000	241/100,000 0.241% (27)	No data available	$20,160	$109MM
Multiple Sclerosis	486,000	149/100,000 0.15% (28)	300200 (26) −284171 (NIH, disease burden 2015)	$21,000	$111MM
Reference population				$7,760

The burden of illness for ME/CFS is more than double that of MS (26), and medical costs for ME/CFS in this study are double those for either Lupus or MS and four times higher than for the general insured (reference) population.

An additional point of comparison is the amount spent on research for these diseases by the National Institutes of Health. Looking at these comparisons for prevalence, burden of illness and annual medical cost, and the amount of NIH funding for these three similar diseases, ME/CFS, lupus, and multiple sclerosis, it is evident that research on ME/CFS is grossly underfunded ($15 vs. $109–111 MM), a point also made by Dimmock et al. (26).

### Limitations of This Type of Study

This study, based on claims data including Medicare and commercial insurance, does not assess the prevalence in Medicaid recipients or the uninsured, two groups in which the prevalence of ME/CFS might be higher, as indicated by community-based studies (19). Furthermore, the financial impact of disability from ME/CFS may lead to Medicaid eligibility, thus removing some ME/CFS patients from the commercially insured population.

Since we were not able to validate ME/CFS diagnosis using codes for some (but not all) of the core symptoms, our machine learning model results must be considered preliminary.

ICD codes used for ME/CFS as of 2018 are inexact and may be applied to individuals with other conditions (see Appendix 1 in Supplementary Material). This introduces an unknown degree of uncertainty to the estimates of prevalence of ME/CFS in this study. In doing this study, we necessarily made a number of assumptions which are stated in the Methods section and are also discussed above. The results might have been different if different assumptions were used. Since this data is from U.S. insurance claims and reflects the practices of U.S. health care providers, these results may not be valid for other countries.

### Implications for the Future and Next Steps

The authors recommend use of the ME diagnosis code (G93.3) rather than CFS (R53.82), which defaults to “Chronic fatigue, unspecified,” for better tracking where symptoms warrant.

The authors also recommend the creation of new symptom codes for post-exertional malaise and substantial impairment in activity levels accompanied by profound fatigue, two of the core symptoms of ME/CFS.

### In Summary

This study is the first to use a large medical claims database to study the characteristics of a large group of individuals who have been diagnosed with ME or CFS and to explore the potential of mining this type of data. This study used a base data set of 50 million individuals tracked over 6 years. The next largest study referenced had a sample size of 90,316, all located in a single municipality.

While the percentage of women diagnosed with ME/CFS is higher than the percentage of men, ME/CFS is not a “women's disease.” Thirty-five to forty percent of diagnosed patients are men.

It is not possible at this time to use symptom codes in medical claims data to identify individuals for whom a diagnosis of ME/CFS might be considered. Introducing new symptom codes for two of the required symptoms identified in the study published by the Institute of Medicine (1) should be considered.

Patients diagnosed with CFS may represent a more heterogeneous group than those diagnosed with ME; this study makes no conclusions about accuracy of diagnosis or quality of care given to ME/CFS patients by providers.

Annual direct medical costs for ME/CFS patients are three to four times higher than average of the reference population and fifty percent higher than for multiple sclerosis or lupus, diseases with similar characteristics.

This study is a “snapshot” and could be repeated in future years for comparison. It would also be interesting to look in more detail at the diagnosis of conditions which are co-morbid with ME/CFS, such as migraine headaches or orthostatic intolerance.

These results show that a prevalence rate 857/100,000 for ME/CFS is not unreasonable; therefore, it is not a rare disease, but in fact a relatively common one.

Based on our results and analysis, ME/CFS should get more attention in research and provider communities, and warrants more education to providers (primary care, specialties, and allied health sciences) to improve the quality of health care and quality of life for affected individuals.

## Author Contributions

Research for this study was carried out by AV, EH, SA, and DK, with direction and support from DP and JA. LB, AD, CL, and PR provided clinical guidance. AD advised on statistics. The manuscript was written primarily by AV, DP, and CP, with editorial suggestions from LB, AD, CL, PR, and JA.

### Conflict of Interest Statement

AV, EH, DK, DP, and JA are employees of UnitedHealth Group. SA is a former employee of UnitedHealth Group. The remaining authors declare that the research was conducted in the absence of any commercial or financial relationships that could be construed as a potential conflict of interest.

## Footnotes

^1^ICD codes for ME and CFS Available online at: https://www.icd10data.com/ICD10CM/Codes/G00-G99/G89-G99/G93-/G93.3 Available online at: https://www.icd10data.com/ICD10CM/Codes/R00-R99/R50-R69/R53-/R53.82 Available online at: http://www.icd9data.com/2014/Volume1/320-389/320-327/323/323.9.htm Available online at: http://www.icd9data.com/2014/Volume1/780-799/780-789/780/780.71.htm

^2^ICD codes related to fatigue Available online at: http://www.icd9data.com/2014/Volume1/780-799/780-789/780/780.79.htm Available online at: https://www.icd10data.com/ICD10CM/Codes/R00-R99/R50-R69/R53-/R53.81 Available online at: https://www.icd10data.com/ICD10CM/Codes/R00-R99/R50-R69/R53-/R53.83
