# Supplementary material for: Estimating Prevalence, Demographics, and Costs of ME/CFS Using Large Scale Medical Claims Data and Machine Learning
Source: Front Pediatr. 2019 Jan 8;6:412. doi: 10.3389/fped.2018.00412 (PMC6331450; doi:10.3389/fped.2018.00412)
Supplement: Supplementary file 1 [file Data_Sheet_1.docx]

**Appendix 1, Interrelationships of ICD codes used for Chronic Fatigue Syndrome (CFS) and Myalgic Encephalomyelitis (ME) in the U.S. as of October, 2018.**

**Chronic Fatigue Syndrome (CFS)**

ICD 9-CM, 780.71, Chronic Fatigue Syndrome.

It converts directly to [R53.82](http://www.icd10data.com/ICD10CM/Codes/R00-R99/R50-R69/R53-/R53.82) Chronic fatigue, unspecified in ICD-10-CM.

In ICD-10 there is no diagnosis code for Chronic Fatigue Syndrome.

In ICD 10, R53.82 is labelled Chronic fatigue, unspecified, and excludes postviral fatigue syndrome ([G93.3](https://www.icd10data.com/ICD10CM/Codes/G00-G99/G89-G99/G93-/G93.3)). Chronic Fatigue Syndrome is listed as an Approximate Synonym.

Clinical information is the same as listed under G93.3, Postviral Fatigue Syndrome, in ICD-10.

**Myalgic Encephalomyelitis (ME)**

 ICD-9-CM 323.9, Unspecified causes of encephalitis, myelitis, and encephalomyelitis.

The term Encephalomyelitis (chronic) (granulomatous) (myalgic, benign) appears in the index only, listed under 323.9

In ICD-10, G93.3 is labelled Postviral fatigue syndrome, is applicable to benign myalgic encephalomyelitis, and excludes chronic fatigue syndrome NOS ([R53.82](https://www.icd10data.com/ICD10CM/Codes/R00-R99/R50-R69/R53-/R53.82))

The clinical information is identical to R53.82.

**Postviral fatigue syndrome**

In ICD-9 there is no entry for Postviral Fatigue Syndrome

Postviral fatigue syndrome is listed as an approximate synonym (one in a long list) for 780.79 Other malaise and fatigue. In ICD-10 789.79 converts directly to G93.3.

**Appendix 2, Proposed Diagnostic Criteria for ME/CFS**

Figure 6. Proposed diagnostic criteria for ME/CFS (Institute of Medicine, 2015)


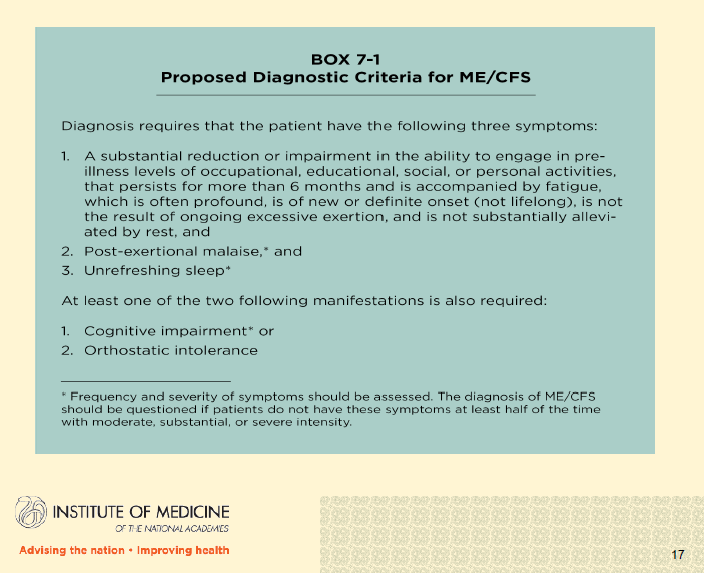


**Appendix 3, About machine learning**

*Machine learning is a method of data analysis that automates analytical model building. It is a branch of artificial intelligence based on the idea that systems can learn from data, identify patterns and make decisions with minimal human intervention* (SAS Institute^[[1]](#footnote-1)^).

In traditional predictive analytics, humans hypothesize a potential relationship between cause and effect or predictive indicator and ultimate result. Data is then used to prove or disprove that relationship. Once confirmed, an analytic algorithm can be created to run against new data sets to predict results.

Machine learning differs from this approach in that a computer begins with no pre-conceived assumptions and uses one or more approaches to evaluate a large number of potential factors, known as ‘features,’ which might play a predictive role in a specific sought-after outcome. This technique allows for orders of magnitude more potential factors to be evaluated and for predictive relationships to be identified that a human might not think to hypothesize.

Machine learning models are created through a defined series of steps. First, a “training” data set must be acquired. The training data set must contain data on all possible features with a well-defined set of “positive” and “negative” outcomes containing records in a ratio of positive to negative within certain bounds required by the specific model approach. The evaluation of the training data set creates a series of weighting tables against each feature indicating the relative predictive impact of each feature. Once the model is trained, it is run against a “validation” data set, again with a well-defined set of positive and negative outcomes, but with those outcomes stripped from the data. The predictions made by the model on the validation data set are then compared to the actual known outcomes to assess the quality of the model. This process can be iterated multiple times by refining the training data set and even manually adjusting the feature weights in a process known as “tuning” the model. In practice, when data starts with prediction, but true outcomes can be known sometime in the future, these outcomes can be fed back into the model through a process of continuous training to produce an ever-improving model.

Machine learning models are assessed based on their predictive capabilities against validation data sets. The results can roughly be categorized in two areas – specificity and sensitivity. These are calculated by looking at occurrence of false positive predictions, false negative predictions, true positive predictions and true negative predictions per the formulas in Figure 6 below.

Sensitivity – Sensitivity is the ratio of correctly predicted positive observations to all actual positives: Sensitivity = TP/TP+FN

Specificity – Specificity is the ratio of correctly predicted negative observations to the total of all actual negatives: Specificity = TN/TN+FP

Figure 7. Graphic illustrating how specificity and sensitivity are calculated in a machine learning model


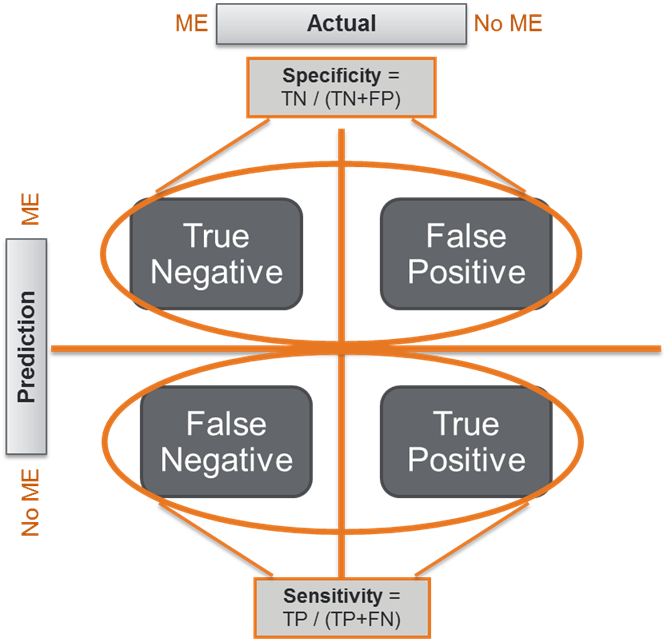


When a model is used against an unknown data set, the confidence in the prediction is one of the outputs of the model. Thus, it is possible to set “thresholds” and produce predictions for true outcomes only when there is a high level of confidence.

**Appendix 4, ICD Codes used in validation of clinical diagnostic criteria**

**Symptom 1 *required**

- ICD9s
  - 78079 Other malaise and fatigue
  - 7993 Debility, unspecified
- ICD10s
  - R53s Malaise and fatigue
    - *Exclude R5382 (Chronic Fatigue Syndrome)*

**Symptom 2: post exertional malaise *required**

- ICD9s
  - E9272 Excessive physical exertion
  - 9945 Exhaustion due to excessive exertion
- ICD10s
  - T733 Exhaustion due to excessive exertion

**Symptom 3: sleep abnormalities *required**

- ICD9s
  - 78054 Hypersomnia, unspecified
  - 32736 Circadian rhythm sleep disorders
  - *327s sleep disorders*
- ICD10s
  - *G47s sleep disorders*
  - G471s Hypersomnia
  - *F511s Hypersomnia not due to a substance or known physiological condition*
  - G472s Circadian rhythm sleep disorders

**Symptom 4: *only one is required**

- **Cognitive impairment**
  - **ICD9s**
    - *799.51 – 799.55 Frontal lobe and executive function deficit, Visuospatial deficit, Cognitive communication deficit, Attention or concentration deficit*
    - *310.2 Postconcussion syndrome*
    - *310.9 Unspecified nonpsychotic mental disorder following organic brain damage*
    - *333.1 Essential and other specified forms of tremor*
    - *333.2 Myoclonus*
    - *337.00 Idiopathic peripheral autonomic neuropathy, unspecified*
    - *337.09 Other idiopathic peripheral autonomic neuropathy*
    - *337.9 Unspecified disorder of autonomic nervous system*
    - *386.10 Peripheral vertigo, unspecified*
    - *780.4 Dizziness and giddiness*
    - *780.2 Syncope and collapse*
  - **ICD10s**
    - *R4184s Cognitive deficits, other (use 5 digits if possible)*
    - *F0781 Postconcussional syndrome*
    - *F079 Unspecified personality and behavioral disorder due to physiological condition*
    - *F09 Unspecified mental disorder due to known physiological condition*
    - *G250 Essential tremor*
    - *G253 Myoclonus*
    - *H81s Disorders of vestibular function*

**Symptom 5: *only one is required**

- **Orthostatic intolerance**
  - ICD9s
    - 4580 Orthostatic hypotension
    - 45989 Other disorder of circulatory system
    - *7850 Tachycardia, unspecified*
    - *4272 Paroxysmal tachycardia, unspecified*
  - ICD10s
    - I951 Orthostatic hypotension
    - I998 Other disorder of circulatory system
    - G9009 Disorder of the autonomic nervous system, unspecified
    - G908 Other disorders of the autonomic nervous system (POTS, post-viral POTS)
    - G909 Disorder of the autonomic nervous system, unspecified
    - R42 Dizziness and giddiness
    - R55 Syncope and collapse
    - *R000 Tachycardia, unspecified*
    - *I479 Paroxysmal tachycardia, unspecified*

1. SAS Institute, Inc. (2018). Cary, NC. Machine Learning. What it is and why it matters. www.sas.com.

   Available online at: https://www.sas.com/en_us/insights/analytics/machine-learning.html (Accessed August 15, 2018). [↑](#footnote-ref-1)
